# Behavioural changes in the city: The common black garden ant defends aphids more aggressively in urban environments

**DOI:** 10.1002/ece3.11639

**Published:** 2024-07-03

**Authors:** Hannah Gaber, Florian Ruland, Jonathan M. Jeschke, Maud Bernard‐Verdier

**Affiliations:** ^1^ Department of Biology Ghent University (Ugent) Ghent Belgium; ^2^ Institute of Biology, Freie Universität Berlin (FUB) Berlin Germany; ^3^ Leibniz Institute of Freshwater Ecology and Inland Fisheries (IGB) Berlin Germany; ^4^ Berlin‐Brandenburg Institute of Advanced Biodiversity Research (BBIB) Berlin Germany; ^5^ West Iceland Nature Research Centre Stykkisholmur Iceland

**Keywords:** aphids, behavioural assay, herbivory, parasitoids, protective mutualism, *Tanacetum vulgare*, urbanisation

## Abstract

Urbanisation alters biodiversity patterns and threatens to disrupt mutualistic interactions. Aside from pollination, however, little is known about how mutualisms change in cities. Our study aimed to assess how urbanisation affects the protective mutualism between ants and aphids, investigating potential behavioural changes in mutualistic ants and their implications for aphids in urban environments. To do so, we studied the protective mutualism between the pink tansy aphid (*Metopeurum fuscoviride*) and the black garden ant (*Lasius niger*) along an urbanisation gradient in Berlin, Germany. In nine locations along this gradient, we measured aphid colony dynamics and proxies for parasitism, quantified the investment of ants in tending aphids and conducted behavioural assays to test the aggressiveness of ant responses to a simulated attack on the aphids. We found that aphid colonies flourished and were equally tended by ants across the urbanisation gradient, with a consistent positive density dependence between aphid and ant numbers. However, ants from more urbanised sites responded more aggressively to the simulated attack. Our findings suggest that this protective mutualism is not only maintained in the city, but that ants might even rely more on it and defend it more aggressively, as other food resources may become scarce and more unpredictable with urbanisation. We thereby provide unique insights into this type of mutualism in the city, further diversifying the growing body of work on mutualisms across urbanisation gradients.

## Graphical abstract

This study investigates the impact of urbanisation on the protective mutualism between pink tansy aphids and black garden ants in Berlin, Germany. Despite urbanisation, aphid colonies thrived and were consistently tended by ants, with a positive density dependence between aphid and ant numbers. Moreover, ants from more urbanised areas exhibited increased aggressiveness in responding to simulated attacks, suggesting that this protective mutualism is maintained in the city.

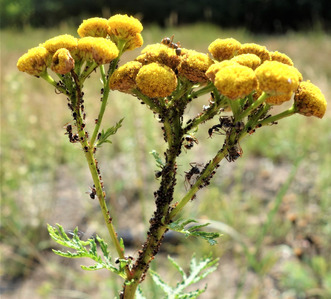

## 1 INTRODUCTION

Human‐induced rapid environmental change leads to biodiversity decline in ecosystems across the globe, not only in terms of species loss (IPBES, 2019) but also through loss and alteration of biotic interactions (Theodorou, 2022; Tylianakis et al., 2008; Valiente‐Banuet et al., 2015). Species that rely on mutualistic interactions for at least parts of their life history are prone to secondary extinctions (Sandor et al., 2022). Over the past decade, severe threats of secondary extinctions have been shown in the disruption of pollination and seed dispersal, two crucial mutualisms for plant reproduction (Fricke et al., 2022; Neuschulz et al., 2016). Protective mutualisms are similarly important to ecological dynamics and seem particularly vulnerable to disturbance by climate change (Blanchard et al., 2021; Mooney et al., 2019), but have remained understudied in the context of urbanisation.

Cities are hot spots of human impact. The urban space is a fast‐growing, heterogeneous mosaic of buildings, road infrastructure and vegetation patches, creating a fragmented matrix of isolated habitat patches for urban species (Putyatina et al., 2017). The distinct microclimate in cities is warmer than their rural surroundings (urban heat‐island effect; Dale & Frank, 2014). It affects plant traits (Cabon et al., 2022) and insect metabolisms (Vucic‐Pestic et al., 2011), and creates potential challenges for herbivores to find their host plants (Schmitt & Burghardt, 2021). In addition, artificial light at night, soil pollution and the use of pesticides and fertilisers in urban gardens and parks generate novel ecological constraints. Altogether, these urban conditions create novel ecosystems and communities, often including a large proportion of exotic introduced species (Kowarik, 2011).

Some insect herbivores, such as aphids, appear to benefit from the new state of biotic interactions in cities via an alteration of both top‐down and bottom‐up controls (Raupp et al., 2010). Aphid performance is expected to increase in urban settings through improved quality and reduced defences of abiotically stressed host plants (plant stress hypothesis; Koricheva et al., 1998; White, 1969), while natural enemies of aphids have been reported to decline in cities (Nelson & Forbes, 2014; Rocha & Fellowes, 2020). Aphid outbreaks in urban green spaces are therefore predicted to become more frequent (Korányi et al., 2021), which in turn could affect their protective mutualism with ants. Ants strongly influence aphid population dynamics (Samuel & Rastogi, 2022; Sanchez et al., 2020), and may thus enhance or down‐regulate herbivory on urban plants. Understanding how mutualistic interactions with aphids change and evolve across urban settings is essential to predicting future dynamics in urban insect communities and mutualistic networks.

Urban ant communities are often dominated by few generalist species (Putyatina et al., 2017) that can take advantage of novel food resources (Penick et al., 2015). Urban environments seem to select for bold, active and opportunistic individuals (Sih et al., 2012; Sol et al., 2013). In the case of urban mutualistic ants, opportunistic strategies may consist of reducing investment in the specialised food‐for‐protection mutualisms with aphids by instead opting for a broader diet. Then again, honeydew of aphids may be an especially valuable resource in cities due to the patchy distribution of vegetation, therefore increasing competition and aggressions between aphid‐tending ant colonies. Urban ants may thus be more aggressive, particularly because aggressiveness and foraging activity are correlated behaviours consistently observed in ants across various contexts (Bengston & Dornhaus, 2015; Blight et al., 2016). However, such typical correlations of behaviours may not persist across urban environments due to different selective pressures: the non‐mutualistic forest ant *Temnothorax nylanderi*, for instance, was found to be less aggressive but more actively foraging in response to urbanisation (Jacquier et al., 2023).

There are different selective pressures in urban habitats acting on ants and aphids, which may influence their mutualistic interaction. Recent studies reported an effect of urbanisation on the intensity (Kremer et al., 2018), diversity (Kök et al., 2022) and outcome (Rocha & Fellowes, 2020) of ant‐aphid mutualisms. The potential changes in ant and aphid traits and behaviour remain largely unknown, however. In the case of plant‐pollinator mutualisms, there is evidence of changes in traits and phenology of the mutualistic partners (Theodorou et al., 2020; Ushimaru et al., 2014), including novel behaviours of pollinators (Ropars et al., 2019). Modification of behaviour is the first and most rapid response of animals to environmental changes (Wong & Candolin, 2015), including in cities (Sol et al., 2013), which raises two questions: Do mutualistic ants change their behaviour in cities? If so, is this change contributing to the success of aphids in urban environments?

We addressed these questions for the mutualism between the pink tansy aphid (*Metopeurum fuscoviride*) and the black garden ant (*Lasius niger*) along an urbanisation gradient in Berlin, Germany. In nine locations along this gradient, we quantified the investment of ants in tending aphids in terms of time, numbers and behaviour, and measured proxies for aphid colony fitness and parasitism. In addition, we recorded the aggressiveness of ant responses to a standardised repeated in situ experiment of simulated attack of the aphid colony.

We tested the following two hypotheses: (H1) Ants in urban areas have access to less habitat and resources than in rural areas, and thus aphidicolous ants are hypothesised to rely more on aphid honeydew as a food source. Based on this hypothesis, we predicted *L. niger* ants working around pink tansy aphid colonies to defend their aphids more aggressively in urban sites within Berlin, and to invest more time in tending duties. (H2) Aphids tend to do well in cities, and we hypothesised that this is also true within a city when comparing different levels of urbanisation. Therefore, we predicted larger pink tansy aphid colonies in the more urban sites within Berlin.

## 2 MATERIALS AND METHODS

### 2.1 Study sites and study system

The study was carried out in Berlin, Germany, in a network of permanent, extensively managed dry grassland patches (i.e. sites) that cover a wide range of urbanisation levels within the city, from old near‐natural clearings in peripheral domanial forests (e.g. Wannsee forest) to wild urban nature areas along roads or railroad tracks (e.g. Nordbahnhof urban nature park) (the CityScapeLab; details in von der Lippe et al., 2020). To quantify the urbanisation level of each site, we used geospatial data to calculate the percentage of sealed (i.e. impervious) surfaces, such as roads and buildings, within a 500‐metre buffer around each grassland patch (hereafter referred to as % sealing; details in von der Lippe et al., 2020). Percentage of impervious surfaces is one of the most common indices of urbanisation (Szulkin et al., 2021), and a 500 m radius is often chosen as a relevant spatial scale (e.g. Gesling et al., 2016).

In May–July 2018, preliminary exploration of 24 grassland sites identified our target system of black garden ant (*Lasius niger* L.) and pink tansy aphid (*Metopeurum fuscoviride* Stroyan) as the most common ant‐aphid mutualism (present in 9 out of 24 sites). We confined our study to these 9 sites, which ranged from 1.5% to 61.6% sealing (see Appendix S1, Figure S1).

*Metopeurum fuscoviride* is a host‐specific aphid species feeding on the common tansy (*Tanacetum vulgare* L., Asteraceae), a native forb growing in open, moderately disturbed habitats around Berlin. It is recognisable by its pink body color and black spot on the abdomen, and produces melezitose‐rich honeydew to attract mutualistic ants (Fischer & Shingleton, 2001). *Lasius niger* is an opportunistic generalist species that often dominates ant communities in urban green spaces (Putyatina et al., 2017) and is frequently found in association with aphids. It is highly attracted to melezitose sugars and selects *M. fuscoviride* as a preferred mutualistic aphid (Fischer et al., 2001). Their mutualism describes a food‐for‐protection relationship: ants offer protection against enemies like parasitoid wasps (Wäckers et al., 2017) and in turn consume honeydew droplets excreted by aphids, which otherwise would accumulate and might foster fungal and parasitic infections (Stadler & Dixon, 2005).

### 2.2 Data collection

**FIGURE 1:**
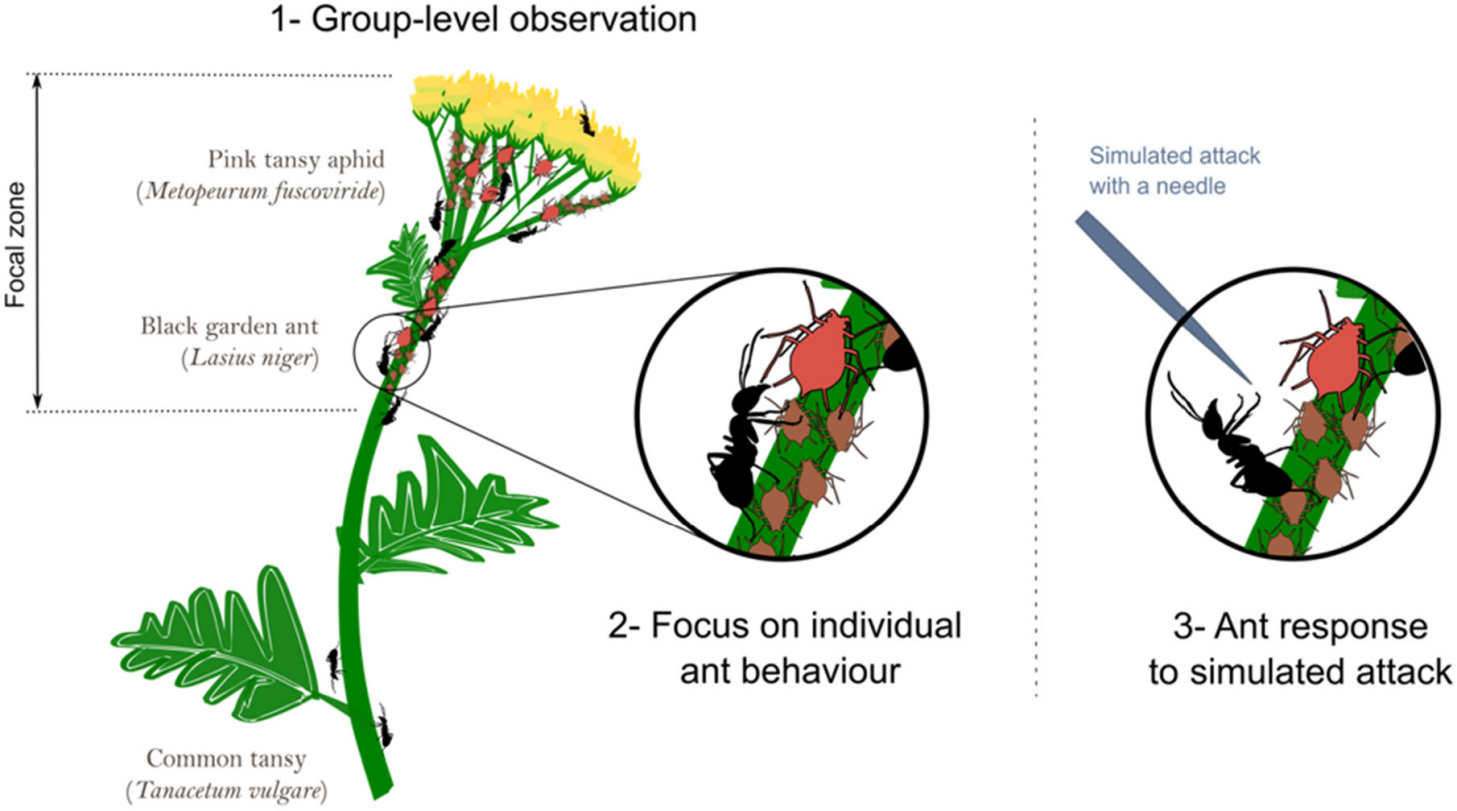
Field assessment of tending and defensive behaviours of the mutualistic ant *Lasius niger* along an urbanisation gradient in Berlin, Germany. Ant behaviour was first observed undisturbed, at the (1) group level and (2) individual level. In a second phase, we (3) recorded behavioural responses of individual ants targeted by a simulated attack using a needle. Each sampling session on a given plant host determined the unit of observation for the study. Observations were replicated along an urbanisation gradient in Berlin and repeated 4–7 times over a month.

The 9 study sites were repeatedly visited between August 1st and September 1, 2018. Each sampling session consisted of visiting a single individual tansy plant infested by a colony of aphids tended by ants (Figure 1), in a given site at a given date. To avoid bias, all observations were made in the morning (9–12 h) and by the same experimenter (HG). Aphid colonies did not persist in all locations throughout the study period, so we carried out 4–7 sampling sessions per site (53 in total). To capture more of the within‐site variation, repeated measurements in the same site were carried out on different host plants where possible, while keeping track of all colony extinctions. In total, we studied 29 aphid‐ant‐*T. vulgare* systems (1–5 systems per study site) of which 13 were repeatedly studied on 2–4 dates. The fieldwork period was characterised by dry and warm weather with temperatures reaching up to 36°C during the sampling sessions. Local temperatures measured during sampling did not vary with % sealing, but they did decrease as the season advanced (Appendix S1, Table S2 and Figure S3). Aphid colonies, typically situated in the inflorescence part of the flowering plant, shifted downwards along the stalk as the flowers started withering.

Each sampling session lasted about 90 min and followed a standard procedure to record (1) ambient temperature (i.e. the plant surface temperature was measured three times – beginning, midpoint and end of the sampling session – in close proximity of the aphid colony using an infrared thermometer), (2) phenological stage of the host plant (budding, flowering, withering or desiccated), (3) ant and aphid numbers, (4) number of parasitised aphids (i.e. aphid mummies) as indicators of parasitoid wasp attacks, and (5) mutualistic ant behaviour.

#### 2.2.1 Ant and aphid numbers

The first observation was done at the group level (Figure 1.1). We started by locating and measuring the plant area with the main aphid cluster, henceforth called ‘focal zone’, a portion of the main stem ranging from 26 to 196 mm in length. We counted all live aphids and mummies (parasitised aphids) in the focal zone. Precise estimation of ant numbers being challenging due to their high mobility in and out of the focal zone, we recorded all ants and their movements into and out of the focal zone during 1 min. This was repeated five times throughout a sampling session. The resulting mean number of ants in the focal zone was used as a proxy for ant abundance. We further calculated the ratio of ant number over aphid number as a first estimate of ant attendance at the group level, hereafter called ‘ants‐per‐aphid ratio’.

#### 2.2.2 Ant behaviour

In between ant counts, we conducted focus behavioural measurements on 2–7 individual ants (Figure 1.2). In each focus session, the behaviour of an individual ant was recorded for 1–5 min (see Appendix S2). Ant individuals selected for these behavioural records were chosen randomly among ants present in the focal zone. Types of behaviour were classified into 5 categories: (1) tending aphids by palpating them with their antennae and/or collecting honeydew; (2) walking around among aphids, on flower heads or leaves; (3) passively being immobile (i.e. inactivity); (4) interacting with other ants by antennae contact and trophallaxis; (5) other behaviours (mainly stretching the abdomen and self‐cleaning). Depending on their behaviour, each focal ant was subsequently sorted into one of three classes based on an existing scheme by Novgorodova (2015): the ‘caretakers’ stayed close to the aphids in the focal zone, the ‘scouts’ left the focal zone to explore other plant parts and the ‘transporters’ left the plant, likely to carry the ingested honeydew back to the ant nest. These focus sessions allowed us to calculate a second estimate of ant attendance, defined as the proportion of time spent by a typical caretaker in tending aphids during the sampling session (*N* = 53; hereafter referred to as ‘tending time’; derived from 172 behavioural records of caretakers by calculating the cumulative recorded time of caretakers spent in tending aphids divided by the total recorded time of caretakers, see Appendix S2 for a detailed description). Data of scouts (*N* = 95) and transporters (*N* = 22) were considered separately, as we did not observe a clear allocation of these ants in aphid care and assumed that these ants may have other assignments in the ant colony.

Finally, we assessed the aggressiveness of ants in response to an intruder by simulating an attack (Figure 1.3). At the end of each session, 2–10 individual ants were randomly chosen among (a) caretakers, actively tending aphids in the focal zone and (b) non‐caretakers doing other tasks outside the focal zone. Note that, as we did not mark individual ants, ants identified as caretakers here were not necessarily all the same individuals as the caretakers identified in the focus observations described above. To simulate the attack, we employed a long needle that was slowly approached and placed point first, a few millimetres from the ant. We categorised 379 ant reactions towards the needle following a slightly modified classification of ant behaviour made by Novgorodova (2015; details of our behaviour classification are found in Appendix S1, Table S4). During the field experiments, we recorded 47 cases where we estimated that our simulated attack failed, as the ants did not recognise the needle as a threat (and either ignored – neutral reaction, ants do not react – or explored it – walking normally on the needle, investigation with antennae). These cases, categorised as ‘no threat reactions’, were treated separately in our data analysis (details in section below). Of the remaining 332 cases, we recorded whether the ant (0) actively avoided contact with the needle – dropping down/running away, or adopting an alerted posture –, or (1) attacked the needle – biting/lunging at the needle. In this way, we obtained a binary estimate of ant aggressiveness.

### 2.3 Statistical analysis

Trends in insect abundance, aphid parasitism, ant attendance and ant behaviour along the urbanisation gradient were analysed using linear (LMM) and generalised mixed (GLMM) models fitted by Maximum Likelihood using the R packages ‘lme4’ (Bates et al., 2015) and ‘glmmTMB’ (Brooks et al., 2017). To mitigate collinearity among predictors, we initially assessed correlations among variables. As a result, temperature and host plant phenology were omitted from the models due to their significant correlation with date (Figure S3). Continuous predictors were then standardised (mean = 0, SD = 1) to improve convergence of the fitting algorithm. Model assumptions were checked using the simulation‐based diagnostic of the ‘DHARMa’‐package (Hartig, 2022) and variables were transformed when needed. We computed Pseudo‐R^2^ to assess model fit (‘MuMIn’; Bartoń, 2022, Nakagawa & Schielzeth, 2013) as well as partial *R* ^2^ to compare variance explained among predictors (‘r2glmm’; Jaeger, 2017). Statistical significance was determined with Satterthwaite's method using *t*‐tests (LMMs) and Wald tests (GLMMs). All analyses were carried out using R version 4.3.2 (R Core Team, 2020).

#### 2.3.1 Ant and aphid abundances

Ant abundance was modelled using the log‐transformed average count of ants in the focal zone at each sampling session. Fixed explanatory factors included urbanisation (% sealing), date and aphid number as well as their 2‐way interactions, and host plant was included as a random effect to account for repeated measurements. Aphid densities were measured as the number of aphids in the focal zone divided by the length of the focal zone in mm. We modelled square root‐transformed aphid densities with an LMM including date, ant number and urbanisation (% sealing) as fixed effects and host plant as random effect.

#### 2.3.2 Aphid parasitism

Aphid infestations by parasitoid wasps were not ubiquitous, creating data with many zeros (no visible presence of parasitism, i.e. no aphid mummies); therefore, variations along the urbanisation gradient had to be tested using a hurdle model. First, we fitted a binomial GLMM to assess how urbanisation influenced the presence or absence of visible parasitism within aphid colonies at each date. Second, after excluding observations with absence of parasitism, we modelled the number of mummified aphids at each date using a Poisson GLMM. The models were implemented using the R‐package ‘glmmTMB’ (Brooks et al., 2017), with predictors including date, aphid number, % sealing, all two‐way interactions, and host plant as random effect.

#### 2.3.3 Ant attendance

Aphid tending effort was quantified in two ways: (1) the ants‐per‐aphid ratio per sampling session and (2) the tending time of individual caretakers per sampling session (Appendix S2). Ants‐per‐aphid ratios were modelled using an LMM on log‐transformed values, while tending times were modelled using a beta regression model (Beta GLMM from the package ‘glmmTMB’, Brooks et al., 2017). Models included date, aphid number and % sealing and all two‐way interactions as fixed predictors, and host plant as random effect.

#### 2.3.4 Ant aggressiveness

We explored effects of urbanisation (% sealing), aphid colony size (aphid number) and date on ant aggressiveness. We assumed that ant aggressiveness might differ between ants involved in tending aphids and ants doing other tasks (context). On the subset of ants for which the simulated attack was considered successful (*n* = 332), we fit a binomial GLMM (with logit link function) with a nested random effect structure of ‘host plant/date’:

$$\begin{array}{c} \text{Aggressive reaction}∼\text{Context}\;\left(\text{tending aphids}/\text{other}\right)+\text{date}\\ +\text{aphid number}+\%\text{sealing}+\text{context}:\text{date}+\text{context}:\text{aphid number}\\ +\text{context}:\%\text{sealing}+\text{date}:\text{aphid number}+\text{date}:\%\text{sealing}\\ +\text{aphid number}:\%\text{sealing}+\left(1,|,\text{host plant}/\text{date}\right) \end{array}$$

We also tested how the likelihood of the simulated attack failing (i.e. not being recognised as a threat) varied along the urbanisation gradient. We fitted a binomial GLMM with the same model structure as the aggressive ant reaction model, using a binary response: (1) ants exhibit ‘no threat reaction’, as opposed to (0) ants reacting with avoidance/aggressiveness to the simulated attack.

## 3 RESULTS

### 3.1 Ant and aphid abundances along the urbanisation gradient

**FIGURE 2:**
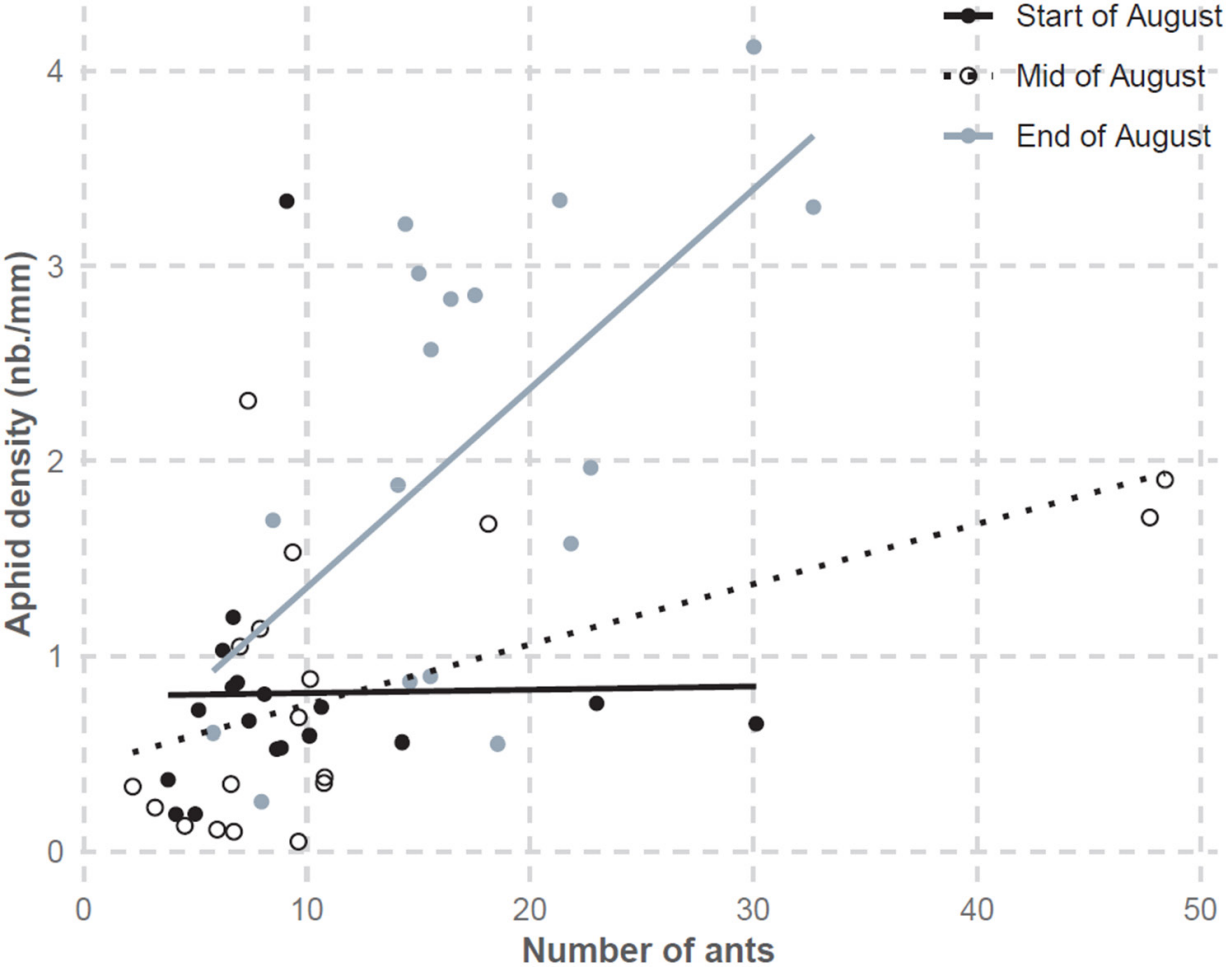
Positive correlation between aphid density and the number of attending ants shown for 3 periods of the field survey (start: 1–11.08.2018; mid: 12–21.08.2018; end: 22.08–1.09.2018). Aphids were counted within an area of the host plant (focal zone) to derive aphid densities (number of individuals/vertical length of the focal zone). Ant numbers are the average number of ants in the focal zone (in decimals, based on 5 counts within 1 min each where ant movement in and out of the zone was monitored). Shown here are the real data and three linear regressions that illustrate the positive interaction between date and numbers of ants and aphids.

The number of ants in the focal zone increased with aphid colony size (Table 1, model 1 and Figure 2) but was not related to % sealing or to the date. As expected during the growing season, aphid densities increased over time (Table 1, model 2). Aphid densities were also positively correlated to the number of ants, and this positive effect became stronger over time (positive interaction between date and ant numbers; Figure 2). A significant negative interaction between date and % sealing in the model suggests that this aphid colony growth was reduced in urban sites, but a closer look at the data indicates a possible outlier effect. In the most urban site of our gradient, only one aphid colony was recorded, and it happened to crash very early on (Appendix S1, Figure S5). No other colonies were found in this urban site afterwards. Excluding this one aphid colony from the models removed any significant interaction term between date and % sealing (Table S6; Figure S7).

**TABLE 1:** Mixed effect models for ant and aphid numbers and ant behaviour.

Model ID	Response variable	Predictors	Est.	SE	*p*	Df resid.	Partial *R* ^2^	Marginal *R* ^2^	Conditional *R* ^2^
1	Ant number	Date	0.088	0.078	.265	45.81	0.021 [0–0.158]	.396	.752
		**Aphid number**	**0.349**	**0.111**	**.003**	**45.80**	**0.145 [0.019–0.341]**		
		% Sealing	0.180	0.093	.066	23.38	0.106 [0.006–0.294]		
		Date:aphid number	−0.047	0.094	.618	45.88	0.004 [0–0.110]		
		Date:% sealing	0.084	0.080	.302	44.16	0.016 [0–0.145]		
		Aphid number:% sealing	−0.094	0.075	.220	45.82	0.024 [0–0.165]		
2	Aphid density	**Date**	**0.125**	**0.056**	**.032**	**40.85**	**0.086 [0.002–0.268]**	.467	.691
		**Ant number**	**0.177**	**0.083**	**.043**	**27.00**	**0.094 [0.003–0.278]**		
		% Sealing	−0.007	0.066	.919	11.45	0.000 [0.000–0.094]		
		**Date:ant number**	**0.250**	**0.070**	**<.001**	**45.87**	**0.183 [0.039–0.383]**		
		**Date:% sealing**	**−0.149**	**0.059**	**.016**	**42.82**	**0.097 [0.004–0.282]**		
		Ant number:% sealing	−0.040	0.090	.657	28.37	0.004 [0.000–0.111]		
3	Ant‐per‐aphid ratio	**Date**	**0.322**	**0.083**	**<.001**	**45.94**	**0.196 [0.046–0.396]**	.539	.829
		**Aphid number**	**−0.805**	**0.117**	**<.001**	**45.32**	**0.435 [0.256–0.607]**		
		% Sealing	0.142	0.102	.176	22.85	0.060 [0.001–0.229]		
		Date:aphid number	0.170	0.100	.092	46.00	0.047 [0.000–0.209]		
		Date:% sealing	0.047	0.085	.582	43.22	0.004 [0.000–0.110]		
		Aphid number:% sealing	0.000	0.08	.988	45.90	0.000 [0.000–0.093]		
4	Tending time	**Date**	**0.243**	**0.103**	**.019**	42.00	—	—	—
		Aphid number	0.089	0.116	.443				
		% Sealing	−0.150	0.082	.067				
		Date:aphid number	−0.134	0.094	.154				
		Date:% sealing	−0.005	0.085	.952				
		Aphid number:% sealing	0.030	0.076	.694				
5	Aggressive reaction	**Context**	**1.170**	**0.427**	**.006**	319.00	**0.045 [0.009–0.106]**	.212	.347
		**Date**	**0.707**	**0.306**	**.021**		**0.026 [0.002–0.078]**		
		Aphid number	−0.637	0.392	.104		0.011 [0.000–0.050]		
		**% Sealing**	**0.680**	**0.319**	**.033**		**0.035 [0.004–0.092]**		
		Context:date	−0.482	0.464	.299		0.004 [0.000–0.035]		
		Context:aphid number	0.257	0.403	.524		0.002 [0.000–0.026]		
		Context:% sealing	0.090	0.418	.829		0.000 [0.000–0.022]		
		Date:aphid number	−0.024	0.297	.935		0.001 [0.000–0.023]		
		Date:% sealing	0.210	0.310	.507		0.005 [0.000–0.037]		
		Aphid number:% sealing	−0.258	0.315	.412		0.009 [0.000–0.046]		

*Note*: All models include ‘host plant’ as random factor, except model 5 which includes ‘date’ nested in ‘host plant’. Significant predictors (*p* < .05) are highlighted in bold. (1) LMM on log‐transformed data; (2) LMM on square root‐transformed data; (3) LMM on log‐transformed data: (4) Beta‐regression GLMM. Note that no *R* ^2^ calculations are available for this method; (5) Binomial regression GLMM. (Results of the mixed effect models for presence and number of aphid mummies are reported in Appendix S1, Table S8.)

### 3.2 Aphid parasitism

Aphid mummies (i.e. individuals visibly infested by a parasitoid wasp) were either absent or only present in low proportions. In total, parasitism was observed in 12 out of 29 aphid colonies surveyed, with no significant relationship found between the presence of parasitism and % sealing (*p* = .504; Table S8, model 6a). Within parasitised colonies, maximum counts of aphid mummies ranged from three to 115 per colony, representing 1.6% to 100% of parasitised aphids. On average, these colonies exhibited 24.5 mummies, equivalent to a parasitism rate of 34.3%. Among parasitised colonies, there were no discernible trends in the number of aphid mummies along the urbanisation gradient (*p* = .167), and neither date nor aphid number was significantly related with this response variable (Table S8, model 6b).

### 3.3 Ant attendance

The ants‐per‐aphid ratio was highly sensitive to aphid colony size, which caused a large span of values ranging from 0.055 to 0.963. The ants‐per‐aphid ratio tended to decrease with aphid colony size (Table 1, model 3): very large ratios mainly occurred in tiny aphid colonies of less than 15 individuals that received care by at least 2 and up to 9 ants. This ratio also increased as the season progressed but remained unrelated to % sealing.

The tending time also varied broadly across colonies and increased over time, but was unrelated to % sealing (Table 1, model 4; Figure S9). Caretakers spent on average half of their time tending aphids (cleaning and feeding on honeydew) and generally devoted the other time to walks among the aphids, communication with other ants, trophallaxis and phases of inactivity (Figure S10).

### 3.4 Ant aggressiveness

**FIGURE 3:**
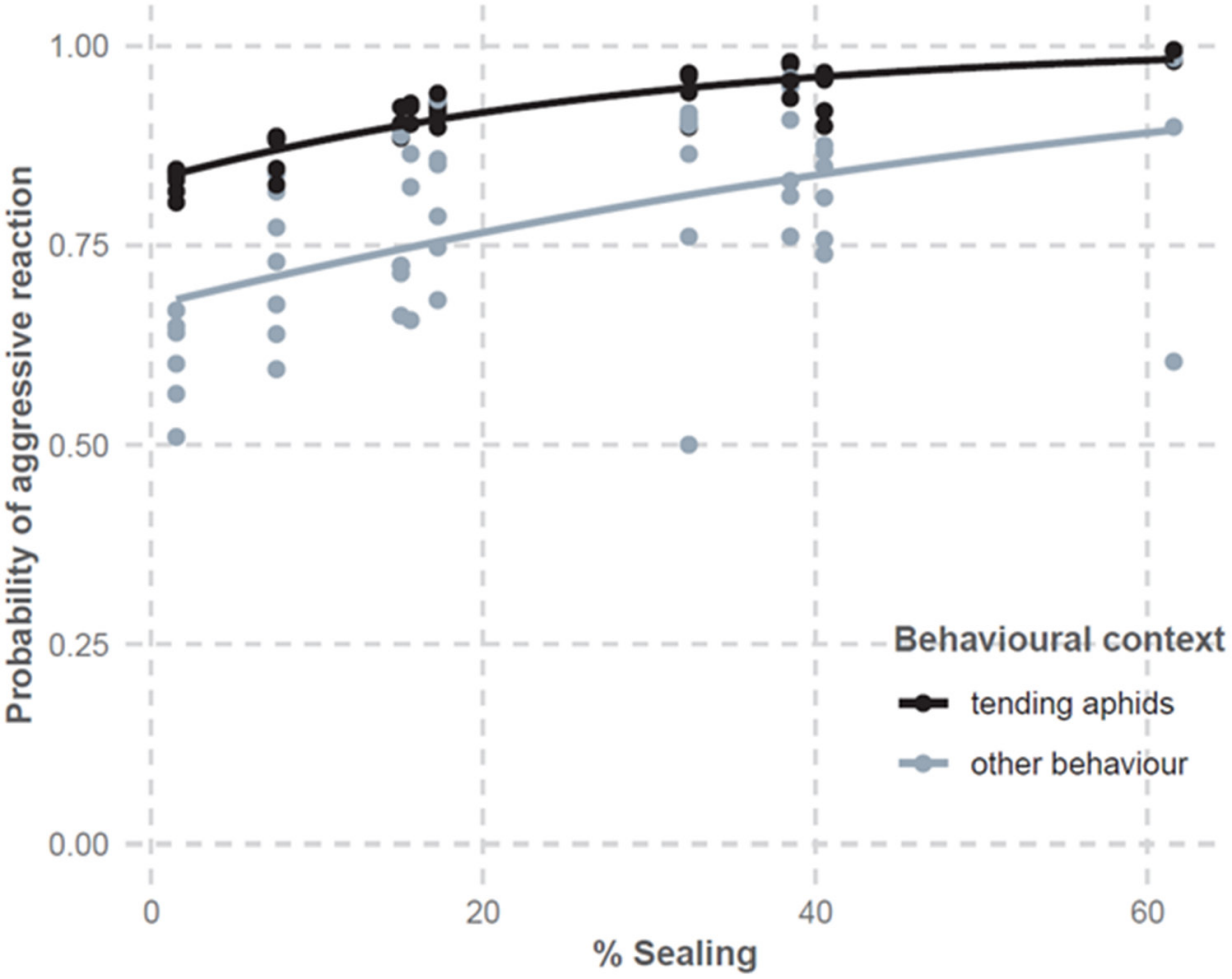
Proportion of ants showing aggressive reactions to a simulated attack along the urbanisation gradient. Dots represent partial residuals and lines correspond to the fit of the binomial GLMM. % sealing is the percentage of sealed surfaces in a 500 m buffer around the study sites. The % sealing variable was standardised to fit the model and back‐transformed to produce the figure.

Ants generally responded aggressively to the simulated attacks. More than 80% of the 332 tested individuals hit the needle with bites, while especially aggressive ones jumped and clung to the needle as we were removing it. Urbanisation levels increased the likelihood of an aggressive ant reaction (Table 1, model 5; Figure 3). Tending ants were more aggressive than ants engaged in other activities (Table 1, model 5; Figure 3), with 90% of caretakers responding aggressively compared to 76% of non‐caretakers. The frequency of aggressive reactions increased over the season (Table 1, model 5). The likelihood of obtaining ‘no threat reactions’ of ants to our simulated attack, considered as failed experiment, was found to be unrelated to % sealing or the behavioural context of the ants, but it did tend to increase over time (Appendix S1, Table S11).

## 4 DISCUSSION

Our results show that ant‐aphid mutualisms appear resilient to urbanisation: over the season, aphid colonies thrived, and mutualistic ants consistently invested time and individuals to tending aphids across the entire urbanisation gradient. Moreover, our study provides a new perspective on how ants adapt their behaviour in urban environments: we found that ants, when in a mutualistic relationship with aphids, display higher aggressiveness in more urbanised areas.

### 4.1 Ant aggressiveness increased with urbanisation

We found support for one specific aspect of our hypothesis, H1, which predicted that *L. niger* ants would defend their pink tansy aphids more aggressively in urban sites within Berlin. High levels of ant aggression have been linked to reduced predation and parasitism in aphid colonies (Buckley & Gullan, 1991), therefore we might expect better‐protected aphids in urban sites, while the ants spend the same amount of time guarding them. However, we found similar rates of parasitism across the urbanisation gradient. This could be due to the parasitoids' refined strategies of oviposition while avoiding ant attack (Hübner & Völkl, 1996; Völkl, 1992). Paradoxically, while more aggressive ants repel predators more efficiently, they actually protect living parasitised aphids, facilitating their mummification (Kaneko, 2003).

Since we simulated the attacks with a needle, the ants' intention behind defending the aphids remains ambiguous. Ant aggressiveness tends to be higher towards competitors than towards aphid enemies (Phillips & Willis, 2005) and, as habitat fragmentation reduces the available foraging area, growing urbanisation likely intensifies competition for finite resources, thus promoting aggressions between rival colonies. Urban night stressors like artificial light and night‐time warming further exacerbate competition by altering species' activity patterns and increasing niche overlap between diurnal, crepuscular and nocturnal species (Tougeron & Sanders, 2023).

Unlike specialised foragers such as *Formica* s. str., which exhibit a clear task division (Novgorodova, 2015), *L. niger* workers in our study seemed to display flexible task allocation. Aphid‐tending ants (‘caretakers’) were more aggressive than other ants, seamlessly combining honeydew collection with aphid protection. While task specialisation is important for improving group‐level performance and productivity among ant workers, flexibility in task allocation is an adaptive alternative strategy, enabling the ant colony to rapidly respond to environmental changes (Loftus et al., 2021). In dynamic urban settings, where labour demands may vary due to frequent disturbances, adaptable task allocation can greatly enhance the ant colony's success. For ants like monomorphic *L. niger*, where pronounced morphological differences are lacking, task flexibility and division of labour are primarily shaped by individual behavioural traits (Wright et al., 2019). The thriving of *L. niger* in urban settings may be attributed to its behavioural plasticity, allowing it to perform various tasks – a trait well‐documented in this ant species. *Lasius niger* workers efficiently handle novel challenges, such as transferring brood in response to sudden nest disturbances (Okrutniak et al., 2023), and colonies demonstrate significant flexibility in reallocating labour, allowing them to quickly and effectively exploit newly available, high‐quality food sources (Bles et al., 2017). Furthermore, *L. niger* displays high behavioural adaptability to novel urban conditions, such as artificial light and increased night‐time temperatures, exhibiting enhanced nocturnal activity and prolonged foraging compared to rural populations (Trigos‐Peral et al., 2024).

Urban ant foraging behaviour is governed by the ‘discovery‐defence’ strategy, meaning that access to a food resource is available to those ants excelling in both rapid discovery and effective defence of the resource (Dáttilo & MacGregor‐Fors, 2021). This aligns with evidence of a behaviour correlation in ants (cf. behavioural syndrome; Sih et al., 2004), linking aggressiveness and exploration (Bengston & Dornhaus, 2015; Blight et al., 2016). Explorative behaviour enhances foraging success of *L. niger* colonies (Pasquier & Grüter, 2016), and among eusocial insects, more aggressive individuals are often better foragers (Lichtenstein et al., 2016; Wray et al., 2011). Consequently, more aggressive and explorative ant individuals are likely better adapted to urban environments, especially for urban foragers engaged in protective mutualisms with aphids, with the same ants being adept at first discovering the honeydew resource, then seamlessly transitioning to tending the aphids while simultaneously defending them against rival colonies.

However, recent research suggests that animal behavioural adaptations to urbanisation vary widely across and within taxonomic groups, rather than following a single trend (Hahs et al., 2023). For instance, urban populations of the forest ant *Temnothorax nylanderi* exhibited reduced aggression compared to rural populations, which contrasts with our findings of higher ant aggressiveness in urban settings. Being non‐mutualistic, *T. nylanderi* may prioritise covering a wide foraging area to maximise food yield. The energy otherwise spent in aggressive behaviour is instead put into more active foraging in urban *T. nylanderi* compared to rural populations (Jacquier et al., 2023). Mutualists, such as *L. niger*, may in turn focus more on defending and securing their access to honeydew resource. Understanding variation in aggressive trait in ants is complex, related to a complex interplay of habitat features and colony characteristics (Maák et al., 2021), emphasising that there is no one‐size‐fits‐all solution to predict changes in ant aggressive behaviour in urban environments. Ants exhibit behavioural adaptations to urbanisation that are highly context‐dependent and shaped by their ecological niches, their evolutionary history, and local environmental conditions.

In particular, the local abiotic environment can influence *L. niger*'s aggressive behaviour, beyond the impact of competitors and natural enemies. The nitrogen content of soils, for instance, was found to positively correlate with ant aggressiveness, suggesting that a nitrogen‐rich diet may stimulate aggression in ants (Krapf et al., 2023). High availability of carbohydrates in the habitat has also been associated with decreased aggressiveness in soil‐foraging ants (Chinarelli et al., 2020). Conversely, mutualistic ants engaged in a protective mutualism with barrel cacti have shown increased protective behaviour and aggressions against predators when provided with enhanced access to carbohydrates (Ness et al., 2009). These findings raise mixed predictions about the potential effects of honeydew, a resource rich in sugars and amino acids, on ant aggressive behaviour. Similarly, ant aggression levels are modulated along temperature gradients by a complex set of factors which are not easily determined (Krapf et al., 2023; Segev et al., 2017). The plant surface temperature we measured at each sampling session did not show a trend in response to urbanisation (Table S2), thus the changes in ant behaviour we observed along the gradient are unlikely to be simple plastic responses to local temperature. However, we observed a decline in temperature over the season, coinciding with an increase in ant aggressiveness. This pattern aligns with recent findings suggesting that *L. niger*'s aggressive behaviour can consistently vary among colonies, while being plastic at the same time, influenced by temperature and season (Menges et al., 2023).

It is important to mention here that our date variable was correlated with other parameters. Specifically, we observed a correlation between date and host‐plant phenology, wherein our studied plants exhibited increasing withering and desiccation as the season progressed (Figure S3). Additionally, there was a positive relationship between date and aphid densities, and between date and proxies of ant attendance (ant‐per‐aphid ratio and tending time increased over time; Table 1). These factors need to be considered when evaluating the temporal increase in ant aggressiveness over the season, as they may potentially drive this pattern instead of the season itself. Host plant phenology and aphid number may affect ant aggression levels by impacting both resource quality (changes in honeydew composition) and quantity (growth of aphid colonies), parameters known to relate with ant aggressiveness (Chinarelli et al., 2020; Pacelhe et al., 2019), and also with ant attendance (Fischer et al., 2002). By contrast, our simulated attacks with a needle tended to be less often successful over time, suggesting an increasing response threshold, possibly via a type of habituation to the experimental protocol, or due to other factors changing over time here as well. Furthermore, since we conducted our study only during summer, it is unclear whether the observed increase in *L. niger*'s aggressiveness along the urbanisation gradient might be a temporally consistent behaviour pattern. Repeating behavioural assays on the same ant colonies during different seasons could provide important insights on how urbanisation affects the outcome of ant personality traits.

### 4.2 Ant attendance did not decline with urbanisation

The second aspect of our hypothesis H1, anticipating higher investment of urban ants in tending duties, was not supported, as ant colonies spent equal per‐capita numbers of workers and time in tending aphids along the urbanisation gradient. The importance of honeydew for the diet of *L. niger* and its effort in maintaining the aphid resource therefore seems to persist across different levels of urbanisation. For an opportunistic forager such as *L. niger*, the stability of a mutualistic association such as this one relies on its partner maintaining a competitive high‐quality food supply (Bshary & Bronstein, 2004). *Metopeurum fuscoviride* is known to be able to actively upregulate the energy content and quantity of honeydew to outcompete other aphid species for mutualistic ant services (Fischer et al., 2001). This adaptation may stabilise the relationship with opportunistic *L. niger* ants across variable environments.

We noted increased ant investment in aphid care as the season progressed. While ants generally exhibit decreased foraging activity as the season advances due to changes in climate variables, vegetation, and resource availability (Bernstein, 1979; Lasmar et al., 2021), our observations do not contradict this seasonal pattern. We only conducted our study during the summer until September 1st, and ant activity would likely have declined further towards autumn as aphid colonies became scarcer. The increasing time investment in tending aphids we observed over the study period may reflect a higher nutritional demand for carbohydrates by ants as the summer progresses. The protein and carbohydrate preferences of ants shift over the season and determine ant foraging decisions (Cook et al., 2011; Novgorodova, 2015). During brood‐caring season, ants maintain a protein‐biased diet to feed the larvae and then switch their preferences to carbohydrate‐rich food, such as honeydew, as the brood grows to adult workers (Stahlschmidt & Johnson, 2018). In addition, host plant desiccation as the summer progresses, and a concomitant change in the nutritional value of phloem, may improve honeydew quality, and ant attendance is known to correlate with this parameter (Fischer et al., 2002). Advancing plant phenology in itself might also cause an alteration of honeydew composition, with changes in amino acid and nitrogen content of honeydew reported at different plant developmental stages for Tuberolachnus salignus (Mittler, 1958). While understanding such temporal changes in resource quality and foraging strategies would require further analyses of honeydew content in the field, our results indicate no obvious difference in these temporal dynamics between less and more urbanised environments.

### 4.3 Aphids thrived across the whole urbanisation gradient

Our hypothesis H2 was not supported, as aphid colony size did not increase with urbanisation. Generally, aphid colonies were healthy, showed low proportions of parasitism and a positive population growth at almost all sites during the survey. The unusually warm and dry weather conditions during the period of fieldwork may have benefitted aphid growth rates, as drought and desiccation of host plants alters phloem transport (Savage et al., 2016), and this may have increased phloem sugar content and food quality.

However, the dynamics of aphid colonies were inconsistent across sites. Notably, our most urban colony collapsed early, and we did not observe any new colony at the site afterwards. Subtle environmental differences among habitats may lead to different outcomes of aphid population dynamics, especially in urban areas (Andrade et al., 2017). Particularly, habitat fragmentation creates dispersal barriers which can reduce the aphid population size at larger scale (Fletcher et al., 2018), thus making the population less resilient against stochastic extinction events of aphid colonies. Furthermore, urban green spaces are often exposed to recurrent and heavy physical disturbances, including pulsed water treatment, which may drive local extinctions of urban aphid colonies. Our most urban study site, for instance, was located in an urban park, along a path frequented by pedestrians, cyclists and dogs, which may explain the early collapse we observed.

Frequent and seemingly random colony collapses are a common feature of natural aphid population dynamics, and during our study, several aphid colonies collapsed along the urbanisation gradient (Figure S5). Predation and parasitism are driving forces behind such collapses (Senft et al., 2017). Contrary to previous observations that urban areas may offer escape from natural enemies (Raupp et al., 2010; Rocha & Fellowes, 2020), we found that aphid colonies were equally threatened by parasitoid wasp attacks across all levels of urbanisation (Table S8). One should mention here that all our sampling sites were located in more or less urbanised areas of the city, hence it is possible that top‐down control by parasitoid wasps would differ at more remote rural or near‐natural sites. This knowledge gap should be covered in future research, as it may give important insights to the debate of declining herbivore predators in cities.

The positive density dependence between ants and aphids we found is in line with other studies (Breton & Addicott, 1992), showing the positive effect of ant numbers (Rocha & Fellowes, 2020) or attendance (El‐Ziady & Kennedy, 1956; Flatt & Weisser, 2000) on aphid growth rates. However, the positive correlation between aphids and ants may alternatively rely on a gradual recruitment of ant workers in response to the growing honeydew resource. Ant foraging strategies are about finding a balance between looking for new food sources and collectively exploiting existing ones (van Oudenhove et al., 2018). Large honeydew resources may thus cause a shift in ant foraging strategy to sustain attendance of aphid colonies, as has been shown in an ant‐treehopper mutualism (Tiong & Morse, 2021). Moreover, *L. niger* foragers are very fast at learning to locate food resources using both route memory and pheromone trails (Grüter et al., 2011), which likely enables the quick recruitment of ant workers to a flourishing aphid colony. Fast and flexible recruitment may promote *L. niger* to thrive in urban environments and together with them their mutualistic partner *M. fuscoviride*.

## 5 CONCLUSIONS

There are cases where environmental change and disturbance have the potential to reinforce, rather than degrade, mutualisms (Kiers et al., 2010). *L. niger* and *M. fuscoviride* intensify their protective mutualism that appears to be resilient to urbanisation. Their mutualistic interaction is associated with a behavioural change in ants in these novel ecosystems. Such behavioural changes, whether resulting from plasticity or evolution, may help urban species like *L. niger* to cope and maintain important biotic interactions in the face of novel challenges in urban environments. The shift towards higher aggressiveness may indicate that mutualist interactions with aphids are becoming more important for *L. niger* in urban environments. Importantly, this shift in ant behaviour might be one of the factors behind the success of *M. fuscoviride* in the city. This raises the question of whether some mutualistic interactions may change towards greater specialisation in cities. Urban mutualisms play an important role in maintaining urban biodiversity. It is therefore essential to continue exploring their diversity beyond pollination and to understand how mutualistic networks evolve in terms of structure and functioning within urban ecosystems.

## AUTHOR CONTRIBUTIONS

**Hannah Gaber:** Conceptualization (equal); formal analysis (lead); investigation (lead); visualization (equal); writing – original draft (lead). **Florian Ruland:** Formal analysis (supporting); supervision (supporting); visualization (supporting); writing – review and editing (equal). **Jonathan M. Jeschke:** Conceptualization (supporting); funding acquisition (lead); resources (lead); supervision (supporting); writing – review and editing (equal). **Maud Bernard‐Verdier:** Conceptualization (equal); formal analysis (supporting); investigation (supporting); supervision (lead); visualization (equal); writing – review and editing (equal).

## CONFLICT OF INTEREST STATEMENT

We declare that there are no conflicts of interest which could influence the results or interpretations presented in this study and all authors confirm the absence of financial, personal or professional relationships that could be perceived as a conflict of interest related to this work.

## STATEMENT ON INCLUSION

All authors were scientists based in the country where the study was carried out. Whenever relevant, literature published by scientists from the region was cited; efforts were made to consider relevant work published in the local language.

## Supporting information


Appendix S1.

Appendix S2.

## Data Availability

The data and code supporting the findings of this study are made openly available in Zenodo at 10.5281/zenodo.12578976.
