# Supplementary material for: Behavioural changes in the city: The common black garden ant defends aphids more aggressively in urban environments
Source: Ecol Evol. 2024 Jul 3;14(7):e11639. doi: 10.1002/ece3.11639 (PMC11221068; doi:10.1002/ece3.11639)
Supplement: Supplementary file 1 — Appendix S1. [file ECE3-14-e11639-s002.docx]

# Supplementary Material I


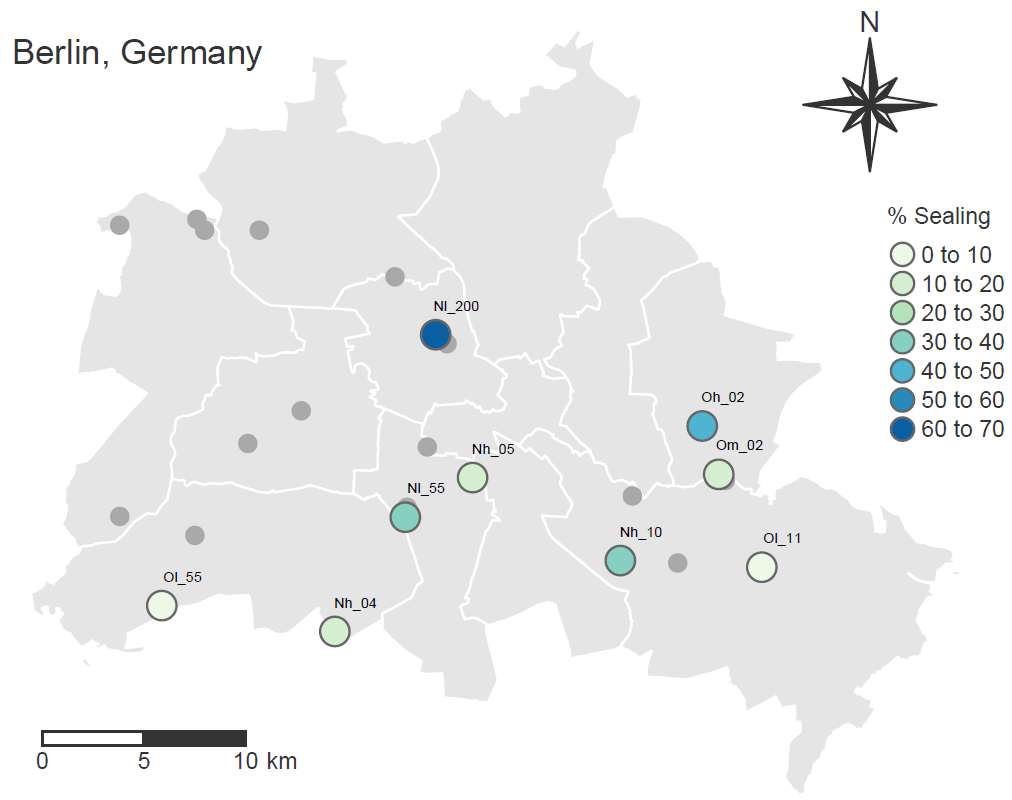


**Figure S1. Map of study sites.** Study sites are located in Berlin, Germany (grey area). We visited a total of 24 sites, 9 of which hosted the same ant-aphid-plant interaction (large coloured circles). The remaining sites were excluded from this study (small grey circles). The colour gradient corresponds to the percentage of sealed surfaces in a 500m buffer around each study site (i.e. % sealing).

**Table S2. Temperature trend along the urbanisation gradient and over the season.** We tested whether the mean temperature per sampling session (average of three plant surface temperature measurements per sampling session) displayed significant patterns along the urbanisation gradient (captured by the percentage of sealed surfaces in a 500m buffer around each study site, i.e. % sealing) and over the season (date). We therefore fit a linear mixed effect model including date, % sealing and their interaction as fixed effects and host plant as random effect. To fit the model, we transformed the response (inverse of the mean temperature per sampling session) and standardized fixed predictors. There was no temperature trend along the urbanisation gradient, but temperature significantly declined as the season progressed (predictor marked in bold). (Note that, since the temperature data was transformed, the model coefficients indicate a reversed relationship between temperature and predictors.)

| **Response variable** | **Predictors** | **Est.**  (*${10}^{-4}$) | **SE**  ($*{10}^{-4}$) | **P** | **Df resid.** | **Partial R²** | **Marginal R²** | **Conditional R²** | |
| --- | --- | --- | --- | --- | --- | --- | --- | --- | --- |
| *Temperature* | **date** | **46.910** | **9.307** | **< 0.001** | **43.16** | **0.340 [0.155-0.534]** | 0.343 | 0.353 | |
|  | **%** sealing | -5.863 | 9.569 | 0.548 | 17.36 | 0.008 [0.000-0.126] |  |  |  |
|  | date:% sealing | 0.993 | 9.633 | 0.918 | 46.84 | 0.000 [0.000-0.098] |  |  |  |


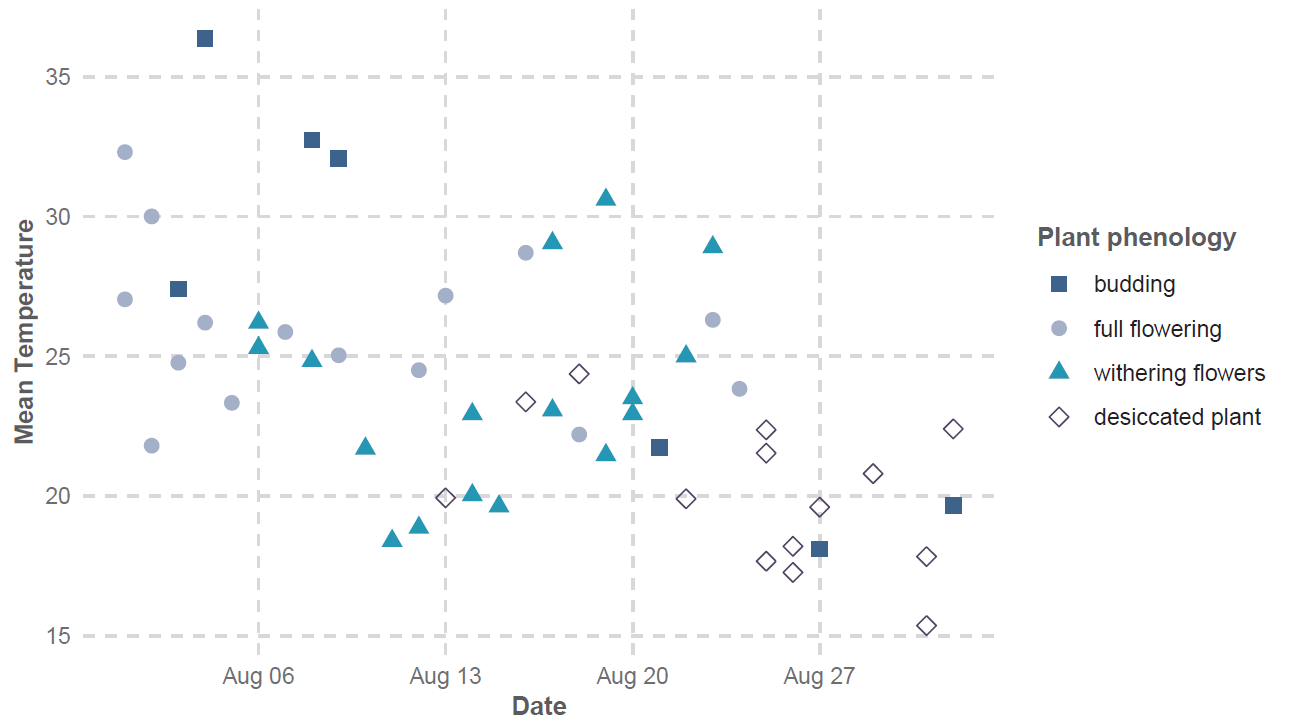


**Figure S3. Variation in** **mean surface leaf temperatures and progression of plant phenology over time.** We observed temporal trends in both host plant phenology and temperature (average temperature per sampling session out of three measurements per session) during the survey periods. The figure depicts the relationships among these two variables and sampling date. To address this collinearity in our data, we retained date and excluded temperature and host plant phenology as predictors in the models of insect abundances, ant attendance, and ant behaviour.

**Table S4.** **Categorization of ant responses to simulated attack with a needle.** We identified seven behaviours in how ants reacted to the simulated attack. We subsequently binned ant responses into two categories (avoidance/aggressiveness) to analyse trends in ant aggressiveness along the urbanisation gradient. Ant responses that could not be clearly categorized as avoidance or aggressiveness (i.e. ‘no threat reaction’) were excluded from the data analysis. These excluded ant responses were evenly distributed across the urbanisation gradient (see below, Table S11). This table provides details of the categorized ant behaviours along with the corresponding number of ants (N) exhibiting each behaviour during our study.

| **Aggressivity score** | **Observed ant behaviour to simulated attack** | **N** |
| --- | --- | --- |
| 1 | “hit-and-run attack” – ant suddenly and quickly attacks the needle, then moves back | 5 |
| *(aggressive response)* | "attack with bites" – ant attacks the needle in a biting/stinging fight | 149 |
|  | "attack jump" – ant lunges at the needle, grasping onto it and biting it | 121 |
| 0 | ant drops down or runs away | 20 |
| *(avoidance)* | "alerted and contact avoidance" – ant stays in place, moving mandibles and/or the abdomen (possibly indicative of its readiness to either attack or flee), while maintaining a safe distance to the needle | 37 |
|  |  |  |
| excluded data (*'no threat reaction’*) | "tolerance, neutral reaction" – ant does not react and ignores the needle | 21 |
|  | "exploration" – ant walks onto the needle, investigation with antennae | 26 |


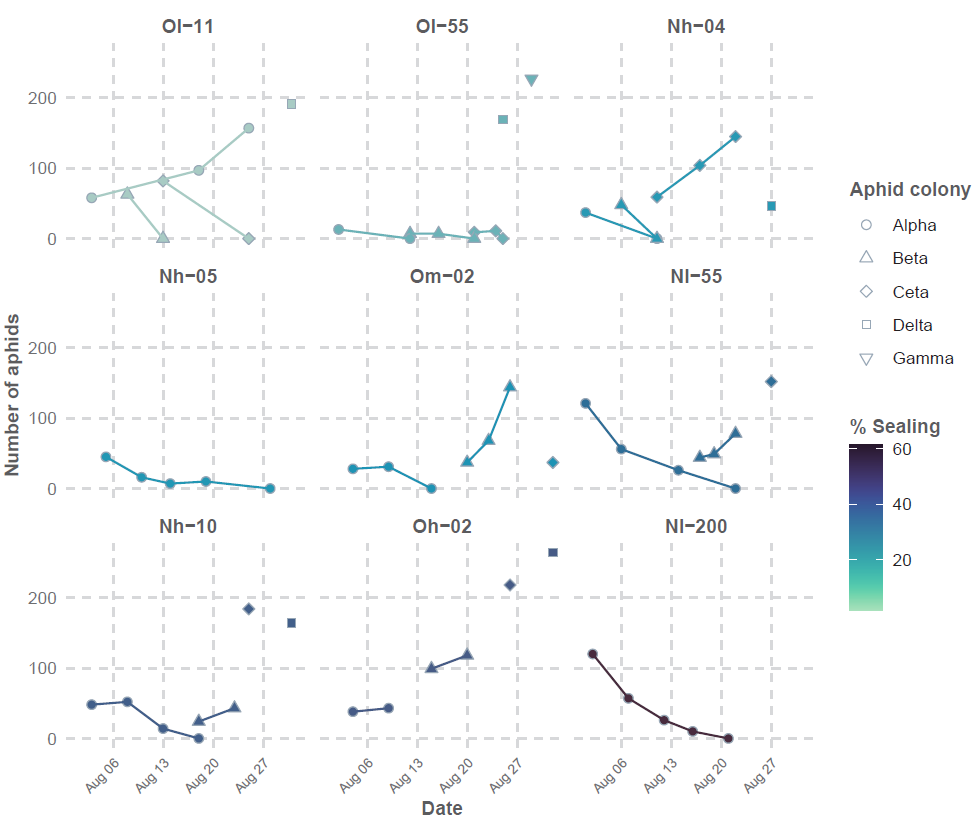


**Figure S5.** **Temporal trends in aphid colony size** (i.e. total number of live aphids) represented for each individual aphid colony (different symbols) in each plot (one per panel). Each panel corresponds to a plot (i.e. site) along the urbanisation gradient (ordered by row from top to bottom according to increasing percentage of sealed areas in a 500m buffer, i.e. % sealing). Local extinctions (all aphids disappeared or mummified by parasitoids) are shown as zero aphids. When possible, multiple colonies were observed in each plot to provide replicates, or to replace extinct colonies.

**Table S6. Model of aphid densities, excluding the most urban plot.** We tested whether the significant ‘date:% sealing’ interaction, which suggested a reduced growth of aphids at urban sites (Table 1, model 2 in the main text), might be an artefact of the early aphid colony crash at the most urban plot. We fitted an alternative linear mixed model of aphid densities that conserved the same structure of fixed and random effects, and where observations at the most urban site were removed (N=4). The interaction was insignificant, suggesting that this weak pattern should be carefully interpreted. Significant variables of the model are marked in bold.

| **Response variable** | **Predictors** | **Est.** | **SE** | **P** | **Df resid.** | **Partial R²** | **Marginal**  **R²** | **Conditional R²** |
| --- | --- | --- | --- | --- | --- | --- | --- | --- |
| *Aphid density* | **date** | **0.170** | **0.050** | **0.001** | **37.74** | **0.198 [0.043-0.407]** | 0.551 | 0.749 |
|  | **ant number** | **0.204** | **0.073** | **0.008** | **34.78** | **0.162 [0.024-0.369]** |  |  |
|  | % sealing | 0.013 | 0.070 | 0.857 | 22.07 | 0.001 [0.000-0.104] |  |  |
|  | **date:ant number** | **0.181** | **0.063** | **0.006** | **41.99** | **0.138 [0.013-0.341]** |  |  |
|  | date:% sealing | -0.010 | 0.064 | 0.876 | 41.79 | 0.000 [0.000-0.102] |  |  |
|  | ant number:% sealing | -0.124 | 0.080 | 0.130 | 32.95 | 0.056 [0.000-0.232] |  |  |


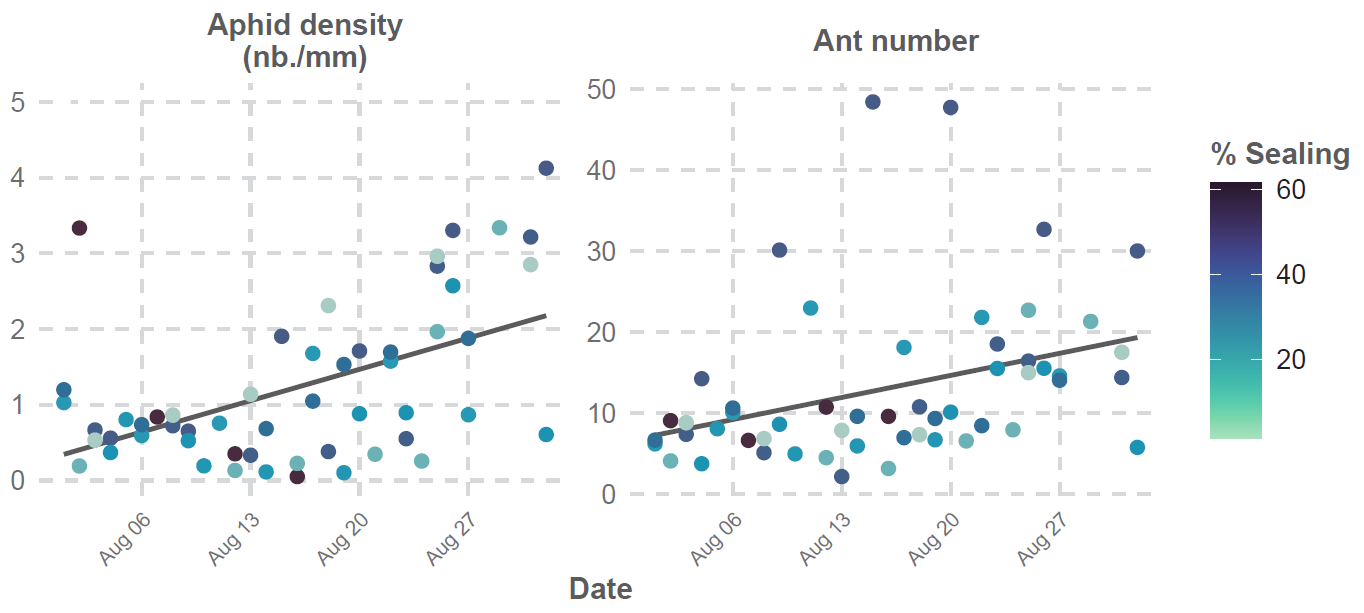


**Figure S7. Increase in aphid densities and ant numbers over the season** Aphid densities are the counts of aphids in the focal zone divided by the length of the focal zone, ant numbers are average counts per sampling session. Dots are coloured according to the percentage of sealed surfaces in a 500m buffer around the study sites (% sealing). Neither response variable was directly related to urbanisation.

**Table S8: Mixed-effect models for presence and counts of aphid mummies**. We analysed our zero-inflated parasitism data with a hurdle model: First, we assessed the effect of urbanisation (% sealing) on presence of parasitized aphids in aphid colonies with a binomial GLMM (model 6a). Secondly, focusing only on these parasitized colonies, we examined trends in the proportion of parasitized aphids along the urbanisation gradient with a poisson GLMM (model 6b). Host plant was included as a random factor. These models were implemented using the R-package ‘glmmTMB’ (Hartig *et al.* 2017).

| **Model ID** | **Response variable** | **Predictors** | **Est.** | **SE** | **P** | **Df resid.** | **Partial R²** | **Marginal R²** | **Conditional R²** |
| --- | --- | --- | --- | --- | --- | --- | --- | --- | --- |
| 6a | *Presence of parasitism* | date | -0.963 | 0.755 | 0.202 | 44 | - | 0.148 | 0.746 |
|  |  | aphid number | -0.596 | 1.106 | 0.590 |  |  |  |  |
|  |  | % sealing | -0.595 | 0.891 | 0.504 |  |  |  |  |
|  |  | date:aphid number | 1.351 | 1.045 | 0.196 |  |  |  |  |
|  |  | date:% sealing | -0.300 | 0.714 | 0.674 |  |  |  |  |
|  |  | aphid number:% sealing | 0.352 | 0.795 | 0.658 |  |  |  |  |
| 6b | *Proportion of aphid mummies* | date | -0.244 | 0.204 | 0.232 | 12 | - | 0.407 | 0.960 |
|  |  | aphid number | 0.197 | 0.293 | 0.501 |  |  |  |  |
|  |  | % sealing | 0.344 | 0.249 | 0.167 |  |  |  |  |
|  |  | date:aphid number | 0.485 | 0.249 | 0.051 |  |  |  |  |
|  |  | date:% sealing | -0.285 | 0.165 | 0.084 |  |  |  |  |
|  |  | aphid number:% sealing | 0.232 | 0.211 | 0.272 |  |  |  |  |


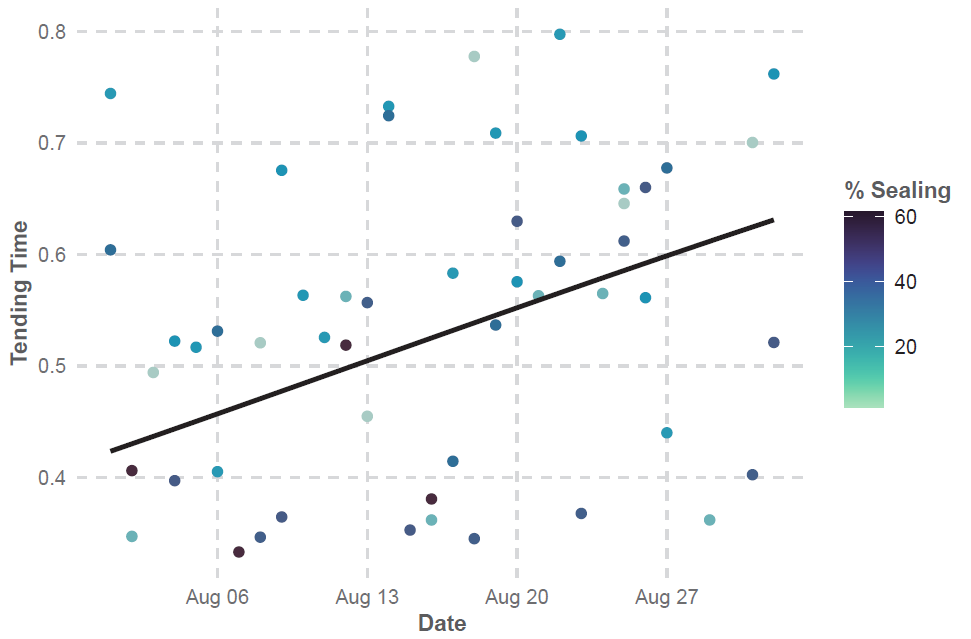


**Figure S9.** **Temporal increase of the proportion of time allocated by ants in tending aphids.** Proportions were derived from field focus observations of individual ants during 2-5 minutes. Dots represent the real data and the line shows the model fit of the Beta GLMM and are coloured according to the percentage of sealed surfaces in a 500m buffer around the study sites (% sealing).


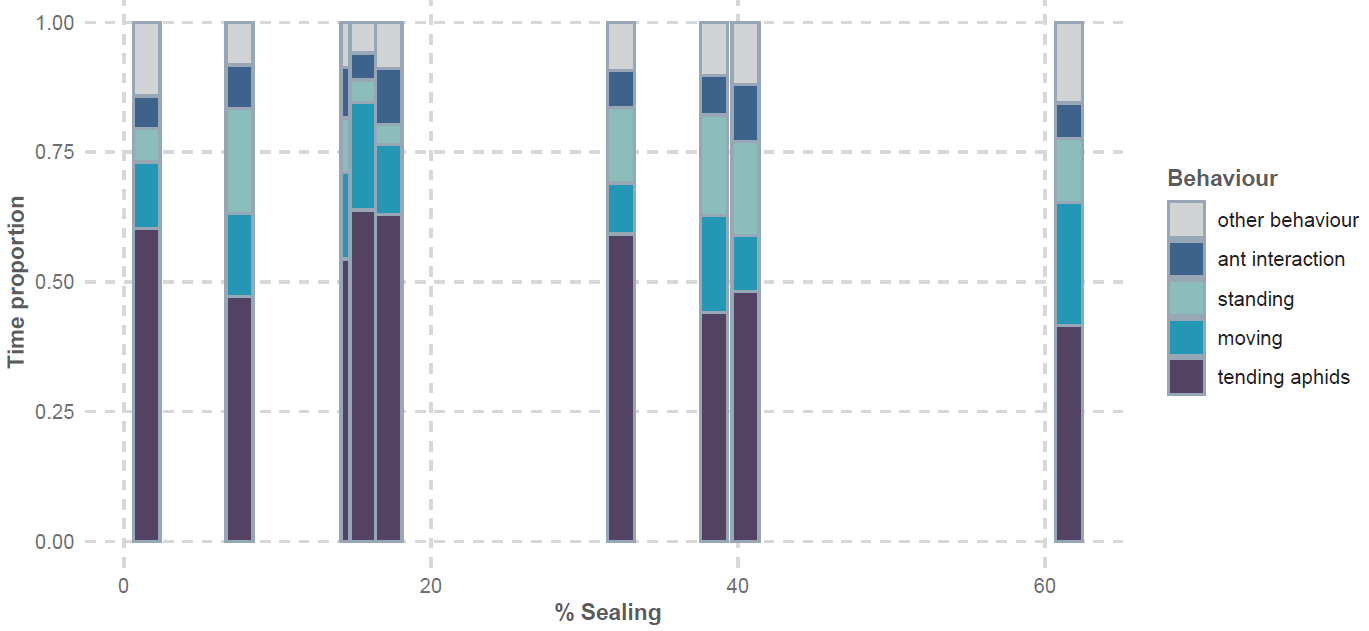


**Figure S10.** **Behavioural time allocation of individual ants along the urbanisation gradient.** Behaviours were classified in five simplified classes based on field focus observations of individual ants during 1-5 minutes. Individual records were averaged across ant replicates per colony, colonies per site, and date of observation (i.e. total time spent by ants in a behaviour/total time of behaviour record). The urbanisation gradient is captured by the percentage of sealed areas in a 500m buffer around the grassland patch. No clear overall trend in time allocation could be detected, except a marginal tendency towards decreased tending time in more urban areas (Table 1, model 4 in the main text).

**Table S11. Mixed-effect model examining the distribution of 'no threat reactions' of ants along the urbanisation gradient.** These responses, characterized by tolerance and explorative ant behaviours towards the simulated needle attack, suggest that ants did not perceive the needle as a threat, and we considered it a failure of our simulated attack. A total of 47 such cases were excluded from our analysis of ant aggressiveness across the urbanisation gradient. To test whether this failure of our simulated attack varied with urbanisation, we tested for variation along the gradient in % sealing using a binomial GLMM with a nested random effect of 'host plant/date'. Results indicate that the likelihood of ants displaying a 'no threat reaction' to the simulated attack, rather than an avoidance/aggressive response, was not related to % sealing. The model was implemented using the R-package 'glmmTMB'.

| **Response variable** | **Predictors** | **Est.** | **SE** | **P** | **Df resid.** | **Partial R²** | **Marginal R²** | **Conditional R²** |
| --- | --- | --- | --- | --- | --- | --- | --- | --- |
| *‘no threat reactions‘ of ants* | Behavioural context | 0.779 | 0.497 | 0.117 | 366 | - | 0.336 | 0.443 |
|  | **date** | **0.900** | **0.311** | **0.004** |  |  |  |  |
|  | aphid number | 0.055 | 0.394 | 0.889 |  |  |  |  |
|  | % sealing | 0.302 | 0.343 | 0.378 |  |  |  |  |
|  | context:date | 0.092 | 0.519 | 0.860 |  |  |  |  |
|  | context:aphid number | 0.730 | 0.611 | 0.232 |  |  |  |  |
|  | context:% sealing | 0.075 | 0.410 | 0.854 |  |  |  |  |
|  | date:aphid number | 0.040 | 0.319 | 0.901 |  |  |  |  |
|  | date:% sealing | 0.218 | 0.290 | 0.451 |  |  |  |  |
|  | aphid number:% sealing | 0.085 | 0.296 | 0.775 |  |  |  |  |
