# Supplementary material for: Behavioural changes in the city: The common black garden ant defends aphids more aggressively in urban environments
Source: Ecol Evol. 2024 Jul 3;14(7):e11639. doi: 10.1002/ece3.11639 (PMC11221068; doi:10.1002/ece3.11639)
Supplement: Supplementary file 2 — Appendix S2. [file ECE3-14-e11639-s001.docx]

# Supplementary Material II

## Calculation of tending time

To estimate tending time, we transcribed all field voice recordings (OLYMPUS Digital voice recorder VN-7000PC) of ‘caretaker’ ant behaviours (2-5 minutes per ant) into a written sequence of behaviours (N=172). The duration of each behaviour in the sequence was estimated in seconds. For behaviours lasting more than a second, we were able to directly read their duration on the device (in seconds) as the time until the next behaviour was recorded. For shorter displays of behaviour (lasting less than a second), we approximated their average duration in a sequence based on the difference between the summed duration of all behaviours lasting more than a second and the total duration of the recorded sequence. The average duration of short elements was thus the time difference in seconds divided by the number of short elements in the sequence. Next, we calculated the total time (in seconds) that each ant had allocated to the 5 predefined activity categories (cf. Methods). We then summed these time periods across all replicate individuals on a given plant at a given date, and calculated the proportion of time allocated to each activity (N=53; sum of time spent by caretakers in activity X / total recorded time of caretakers*;* see Fig. S9).
